# Ultrafast Chemistry of Water Radical Cation, H_2_O^•+^, in Aqueous Solutions

**DOI:** 10.3390/molecules23020244

**Published:** 2018-01-26

**Authors:** Jun Ma, Furong Wang, Mehran Mostafavi

**Affiliations:** Laboratoire de Chimie Physique, CNRS/Université Paris-Sud 11, Bâtiment 349, 91405 Orsay, France; ma.jun.26m@st.kyoto-u.ac.jp (J.M.); furong.wang@u-psud.fr (F.W.)

**Keywords:** water radical cation, electron transfer, oxidizing radicals, radiation chemistry

## Abstract

Oxidation reactions by radicals constitute a very important class of chemical reactions in solution. Radiation Chemistry methods allow producing, in a controlled way, very reactive oxidizing radicals, such as OH^•^, CO_3_^•–^, NO_3_^•^, SO_4_^•–^, and N_3_^•^. Although the radical cation of water, H_2_O^•+^, with a very short lifetime (shorter than 1 ps) is the precursor of these radicals in aqueous solutions, its chemistry is usually known to be limited to the reaction of proton transfer by forming OH^•^ radical. Herein, we stress situations where H_2_O^•+^ undergoes electron transfer reaction in competition with proton transfer.

## 1. Introduction

Liquid water is the key compound for our existence on this planet and it is involved in nearly all biological and chemical processes. The role of contemporary physics and chemistry of liquid water exposed to ionizing radiation (photon, fast electron, X-rays, heavy ions, etc.) is significant in such diverse fields as photon science, radiotherapy, nuclear reactors, radiation chemistry, nuclear waste management, etc. [1,2,3]. Since the discovery of X-rays and natural radioactive phenomena, the chemistry derived from water radiolysis has been studied intensively over the last one hundred years. The interaction of highly energetic photons or charged particles with water initially result in the ejection of a quasi-free electron from the valence shell in general, leaving behind a positively charged radical cation (H_2_O^•+^). The earliest time of H_2_O^•+^ formation is estimated to be on the timescale of attoseconds (10^−18^ s) or subfemtosecond based on the uncertainty relationship (ΔEΔt ≈ *ħ*) [4]. In addition to their recombination, both of these charged species subsequently follow their own pathway of chemical reactivity. The hot electron relaxes into solvent molecules and gets trapped as a hydrated electron (e_hyd_^−^), whilst H_2_O^•+^ rapidly forms oxidizing OH^•^ radical via proton transfer.

(1) $$H_{2}O^{•}{}^{+}+H_{2}O\to OH^{•}+H_{3}O^{+}$$

The chemical framework in which water radiolysis involving OH^•^ and e_hyd_^−^ occur is now well understood experimentally as well as theoretically. The OH^•^ radical is thought to be mainly responsible for much of the radiation-induced oxidation that occurs in dilute aqueous solutions and biological system. Even if this statement is still consistent for an important number of situation, the ultrafast chemistry of its precursor, water hole (H_2_O^•+^) immediately following ionization of liquid water, is a puzzling and very challenging subject in radical chemistry induced by ionizing radiation. The situations where H_2_O^•+^ undergoes ultrafast one-electron oxidation in competition with proton transfer have important practical influences upon nuclear waste storage, nuclear fuel processing and radiotherapy, and remain to be clarified to better assess safety concerns such as the component corrosion and hydrogen emission.

Until now, real-time observation of this species H_2_O^•+^ is still lacking because of the limitation of the time resolution in currently available high energy pulse techniques to typical picosecond time scales. The proton transfer rate constant in the gas phase is about 8 × 10^12^ L mol^−1^ s^−1^ [5]. The extrapolation of this constant by simply taking into account the concentration of water gives an extremely short lifetime which is estimated at approximately twenty femtoseconds, i.e., 18 × 10^−15^ s. Indeed, ab initio molecular dynamic simulations suggested the lifetime of this radical cation in liquid phase is actually less than 40 fs [6]. Alternatively, the chemical reactivity of H_2_O^•+^ was arisen not long after the discovery of hydrated electron in 1962. Hamill et al. [7,8] suggested that holes in water could be trapped prior to hydration using sufficiently high concentrations of solutes such as NaX (X = Cl, Br, F, I) or sulfate salts. The high values for the yields of secondary radicals NO_3_^•^, Cl_2_^•−^ or SO_4_^•−^ from steady-state scavenger or time-resolved nanosecond pulse radiolysis measurements were attributed partially to electron transfer from H_2_O^•+^ radical to anions [7,8,9]. In addition, the water cation radical produced in an inner hydration layer of DNA was considered as a direct-type damage based on the ESR (electron spin resonance) measurements at low temperature [10]. These early studies suggested that the H_2_O^•+^ radical could initiate oxidation processes both in the homogeneous solutions and in interfacial biological systems prior to the generation of OH^•^ radical, but the results are very speculative because the time scales for these reactions are very fast (~100 fs). In addition, as the direct effect of radiation on the solute produces similar chemical species with those produced by the oxidation of the solute by H_2_O^•+^, a peculiarity between these two reactions was not obvious, and the role of H_2_O^•+^ in electron transfer reaction remained controversial.

An oxidizing free radical, for instance, M^•+^ is often involved in the stepwise oxidization of an electron donor via one-electron transfer mechanism. Therefore, the reduction potentials of the couples M^•+^/M is of particular value of predicting the direction of the free-radical redox reactions and in some case the rate constant on the basis of Marcus electron transfer theory. Conventional electrochemical techniques are not applicable to determinate this thermodynamic property of the individual step because the intermediates are short-time lived and they are not easy to be observed. The pulse radiolysis setup consists of an accelerator which delivers a high energy electron pulse to create reactive radicals, or charged species via ionizing or exciting the medium. When coupled with detecting methods such as transient absorption spectroscopy, it allows the production of the transients in a controlled manner and to directly study the redox reactions and relevant physical properties of those intermediates in liquids at various time scales. Therefore, it should be very useful to determinate the redox potential of each individual step. As an example, the DNA nucleotides oxidative reactions or others involving OH^•^ radicals can be investigated by saturating the irradiated solutions with the N_2_O gas to remove the reducing hydrated electrons (e_hyd_^−^ + N_2_O → N_2_ + O^−^) [11]. Additionally, pulse radiolysis techniques can also produce the strong oxidizing SO_4_^•–^ radicals to study the one-electron oxidation reactions when the solution contains appreciate amount of NaS_2_O_8_ salts (e_hyd_^−^ + S_2_O_8_^2−^ → SO_4_^2−^ + SO_4_^•−^) [12,13]. In this case, the redox potential of DNA bases (T, G, A, C) radical cation was subsequently measured, indicating the sites of hole transfer along the DNA duplex and how easily the DNA can be oxidized by free radicals [14,15].

**Figure 1:**
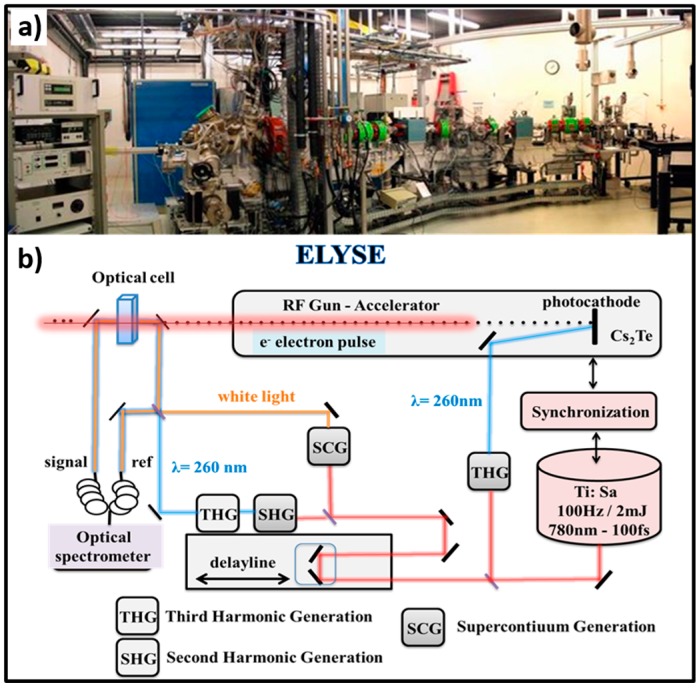
(**a**) Pulse radiolysis facility (unique in Europe) based on the ELYSE picosecond pulsed electron accelerator from the Physical Chemistry laboratory in Orsay, France; (**b**) Schematic description of the synchronizing the electron beam for ionization with a laser beam to probe the species created by the electron pulse. The spectroscopic technique used for this study is the transient absorption based on the pump-probe principle, which makes it possible to overcome the limited temporal resolution of electronic detectors. The setup makes it possible to repeat the measurement for different delays between the pump and the probe and at different wavelengths, and it is thus possible to reconstitute step by step the evolution of the transient processes.

In radiation chemistry, the majority of fast radicals or electrons-induced reactions occur at a diffusion or nearly-diffusion controlled process with a second-order rate constant ranging from 10^9^ to 10^10^ L^−1^ mol s^−1^ under ambient conditions [16,17]. For this reason, over past decades, pulse radiolysis based on a nanosecond electron pulse has been shown to be sufficient to fully resolve numerous radicals’ reactions on their own reaction time. However, as mentioned above, the water radicals cation H_2_O^•+^ proceeds the chemical events much faster than one picosecond and hence a deep understanding of this radical’s chemistry called for a shorter electron pulse. The femtosecond laser-driven accelerator shown in Figure 1 has been recently established at our ELYSE—the facility named after Lysis (Greek for degradation) by Electrons, which achieves a high energy (7–8 MeV) electron beam with a pulse width of 7 ps [18]. The detecting system is based on the transient absorption spectroscopy with a probe light ranging from 380 nm to 1500 nm [19]. Similar picosecond pulse radiolysis facilities have been established over the world, such as Laser Electron Accelerator Facility (LEAF) at Brookhaven National Laboratory, Linear Accelerator (LINAC) facility at Tokyo University, etc., and many of them have reached their full capacity [20,21,22]. Therefore, this unique time resolved technique based on high energy electron pulse enables us, although not ideally, to explore the ultrafast chemical reactivity of H_2_O^•+^ through the scavenging method in a variety of highly concentrated aqueous solutions.

Accordingly, in this review, we present the key findings to show the occurrence of ultrafast electron transfer of H_2_O^•+^ by using highly concentrated solutions. By observing the formation of secondary radicals such as NO_3_^•^, SO_4_^•−^, X_2_^•−^ (X = Cl, Br) or H_2_PO_4_^•^ at picosecond scale, the radiolytic yield of H_2_O^•+^ participating in the reaction as a function of concentration is obtained and isotopic effects have been comparatively studied. Our results imply the chemical situations where the reactivity of H_2_O^•+^ or D_2_O^•+^ plays a more important role in competition with proton transfer to generate OH^•^ or OD^•^ radicals. By further using the diffusion-kinetic simulations of the spur reactions induced by the incident electrons, we established the first semi-quantitative estimation of the H_2_O^•+^ radicals scavenging fractions for a wide range of solutes. In addition, it is suggested that H_2_O^•+^ is the strongest oxidative radical comparing with other oxidants in aqueous solutions based on an estimation of its redox potential value. Finally, it is rationalized that the oxidation of H_2_O^•+^ might be extended to the interface chemistry or biological system which constitutes as a similar water closely trapped environment with that in concentrated solutions.

**Figure 2:**
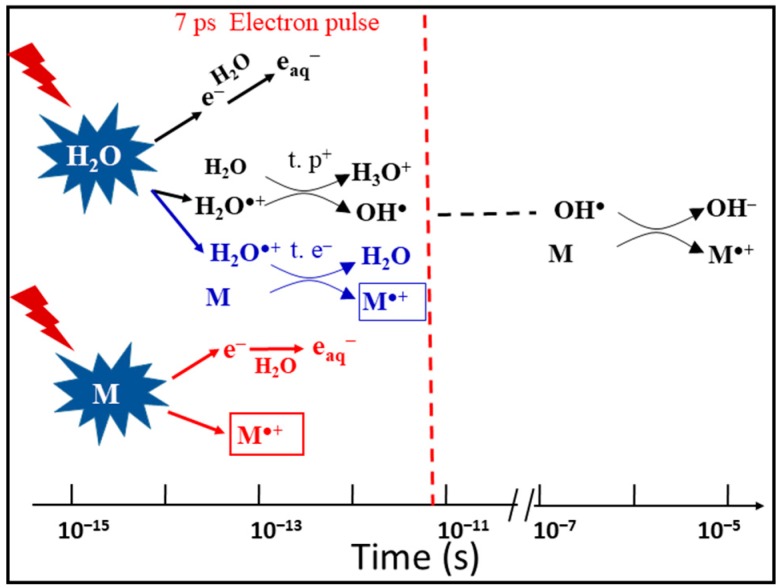
Schematic description of the reactions occurring in solutions containing a solute M at high concentration. At subpicosecond time scales involving proton transfer (t. p^+^, black), direct ionizing of solutes M (red), ultrafast electron transfer (t. e^−^, blue) and electron relaxation. Picosecond electron pulse radiolysis is ready to observe the formation of M^•+^ within the pulse. The oxidation of M by OH^•^ radical takes place at longer time, which does not affect our observation.

The 7 ps pulse radiolysis set-up is used to demonstrate the oxidation reaction of H_2_O^•+^ in different solutions. As the lifetime of the H_2_O^•+^ is too short to be observed directly, the product of the oxidation reaction is observed just after the electron pulse. The oxidation of M by H_2_O^•+^ and by direct effect gives M^•+^ which can be observed. Other oxidation reactions, such as oxidation by OH^•^ radical, are excluded at short time. Therefore, the yield of M^•+^ measured precisely at 7 ps gives us the information about its oxidation by H_2_O^•+^ (Figure 2).

## 2. Oxidation Reactions by Radicals

Oxidation reaction is a very important class of chemical reactions in solution. One example is corrosion, that is to say, the alteration of a material by an oxidant, or oxidative stress which is a type of aggression of the constituents of the cell, involved in many diseases [23,24,25]. Some oxidizing species such as O_2_ are stable, but there are also many other reactive oxidizing species, which are not stable in solution. The very oxidizing free radicals that are commonly known in aqueous solutions include OH^•^, O_2_^•–^, HO_2_^•^, CO_3_^•–^, NO_3_^•^, SO_4_^•–^, HPO_4_^•–^, etc., and can be generated in a radiation chemical procedure [26]. These reactive radical species play a very important role because they act as intermediates in many mechanisms. Free radicals instinctually associated with unpaired electrons in their outer orbitals react with organic or inorganic compounds by various modes. They may abstract a hydrogen atom or add to an unsaturated bond, and they may also reduce or oxidize a substrate via one-electron transfer mechanism. Therefore, the chemistry of free radicals is very rich. In particular, their reactivity is very much studied in the metabolic system of the human being where they can trigger serious diseases [27]. However, some of these radicals can also be used to treat certain diseases, such as cancer using radiation therapy. Because of their very high reactivity, the lifetime of these species is often very short, ranging from milliseconds to picoseconds. Their lifetime depends very much upon their environment. To observe the reactivity of these species, it is necessary to resort to sophisticated experimental setups that allow the monitoring of their reactions, sometimes ultrafast, as a function of time. Thus, to create these radical species, lasers or pulsed electron accelerators are often used, and coupled with a time-resolved detection method via exploiting the optical properties of transient species.

In past decades, numerous studies have been performed in relation with their properties using pulse radiolysis or photolysis methods [28]. The term of oxidizing a molecule means capturing an electron from another molecule. The oxidation force of a species is defined by its redox potential. The redox potential is an empirical quantity expressed in volts and denoted by *E*° for the standard potential under conditions of normal concentration [29]. The values of the redox potential are expressed with respect to the reference potential of the standard or normal hydrogen electrode (NHE) taken equal to zero [2,30]. The higher the value of the redox potential, the more oxidizing the species is. For example, molecular oxygen is an oxidative species and forms O_2_^–^ by capturing an electron of a molecule. The redox potential of the O_2_/O_2_^•–^ couple is −0.33 vs. NHE [31].

**Figure 3:**
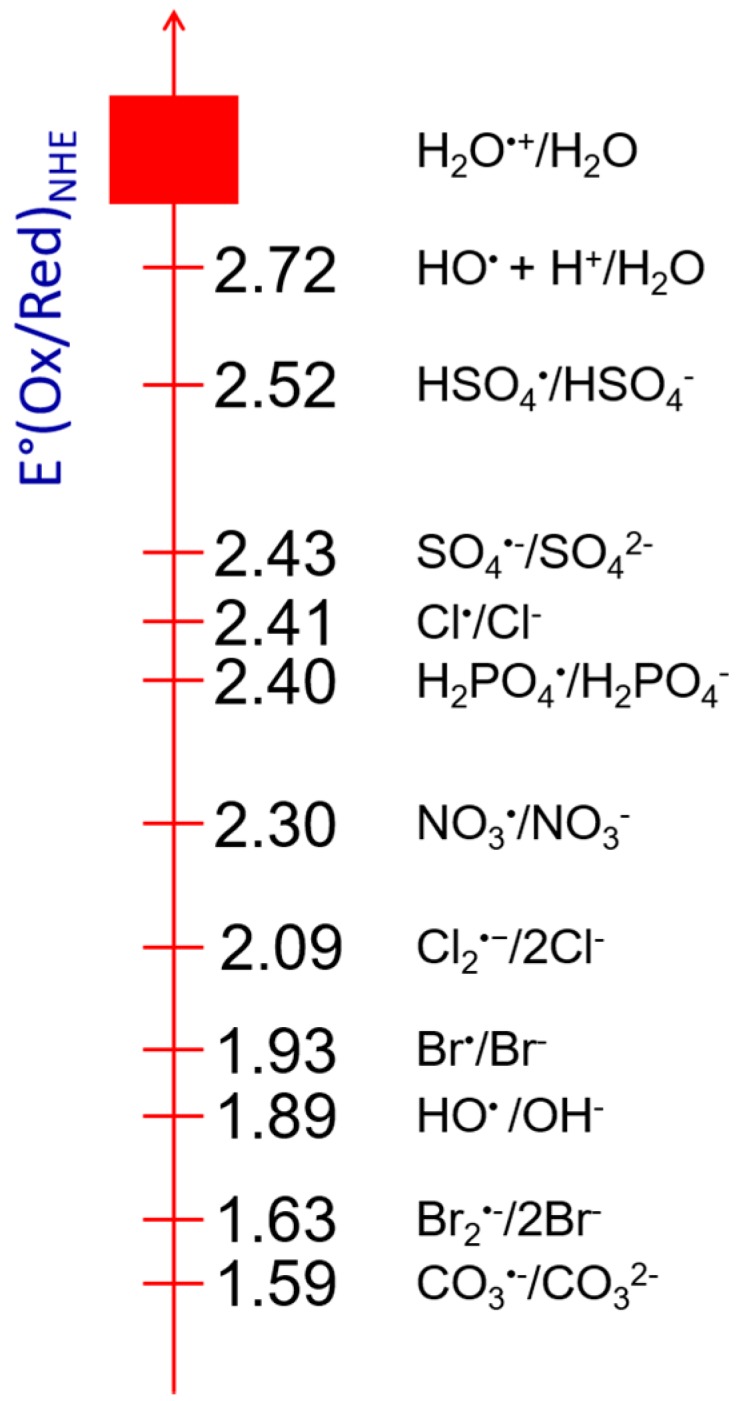
Scale of the values of the redox potential of certain transient radicals with respect to the standard hydrogen electrode (V/NHE).

The value of the redox potential of the transient radical species is reported in Figure 3. It is determined kinetically by pulse techniques according to their ability to oxidize molecules of known potential. In this series, the OH^•^ radical in acid medium is a very strong oxidizing radical in aqueous solutions, comparing with others, such as SO_4_^•–^ (2.52 V) or H_2_PO_4_^•^ (2.40 V) [32]. It is reasonable because OH^•^ radical is able to oxidize the solutes such as SO_4_^2–^, leading to the formation of the secondary radical SO_4_^•–^. In water radiolysis, however, the oxidizing ability of its precursor (H_2_O^•+^) due to its ultrafast proton transfer has not been yet considered. We use a simple thermodynamically cycle for a rough estimation of the redox potential of these radicals. The ionizing potential of a liquid water molecule is measured to be 11.16 eV by liquid-jet photoelectron spectroscopy, which is accordance with the value (11.7 eV) from theoretical predictions by Mozumber [33]. The solvation energy of H_2_O^•+^ is unknown but it is assumed that this energy value is similar with that of H_3_O^+^. In light of this, the standard redox potential of H_2_O^•+^/H_2_O couple is higher than 3 V vs. NHE, showing it is indeed the strongest oxidizing species in liquid water (Figure 3).

Comparing with the high mobility of electrons produced in water radiolysis, the mobility of H_2_O^•+^ is considered to be relatively low, as the infrared spectroscopy study of water cluster radical cations (H_2_O)_n_^•+^ (*n* = 3–11) showed that it is localized and weakly hydrogen bonding with its neighboring water molecules [34]. Its role in previous radiation chemical studies of dilute solutions was completely ignored. In fact, its chemistry strongly depends on the environment and differs greatly from that of OH^•^ radical, which often reacts with a substance via H abstraction or addition instead of one-electron transfer [35]. One may wonder how a radical cation that has such a short life can oxidize another species. Indeed, before reacting, the radical and the species that will be oxidized should diffuse to get closer to each other. However, even if the electron transfer reaction is ultrafast, the diffusion of the reactants through the solvent molecules would require a minimum time (a diffusion limit for the rate constant). For example, the radical OH^•^, which has a very large diffusion coefficient (D = 2 × 10^−9^ m^2^ s^−1^) [17], must nevertheless diffuse during about 1 μs to reach a molecule in solution at a concentration of the order of 10^−4^ M. If the solute is in one molar concentration, this diffusion time is reduced to 60 ps. Under these conditions, the reaction is not likely to take place for the radical cation H_2_O^•+^ because of its immediate proximity with H_2_O and its proton transfer reaction has a lifetime much shorter than the time required for diffusion. Therefore, the only possibility of reaction for this radical is the in situ reaction. That is to say, this radical must be in contact with a target molecule in a concentration of similar magnitude to that of water. Thus, the diffusion is no longer necessary and it can possibly oxidize the molecule which is in its immediate vicinity. The verification of the occurrence of the ultrafast electron transfer of H_2_O^•+^ has initially been made in aqueous NaX (X = Cl, Br) solutions by picosecond pulse radiolysis [36,37,38,39]. In these systems, the energy in proportion to the electrons fraction, is also deposited on the solutes, so the direct ionizing of the solute itself cannot be avoided, resulting in the additional yield of secondary radicals (X^•^ or X_2_^•−^). To make it easier to be understood, it is worth noting that, in radiation-induced chemical reactions, the amount or concentration of radicals generated is associated with the radiation dose. The radiolytic yield or a more specific name *G* value is defined as the number of mole per joule of absorbed dose.

## 3. Reactivity of H_2_O^•+^ in Halide Solutions

**Figure 4:**
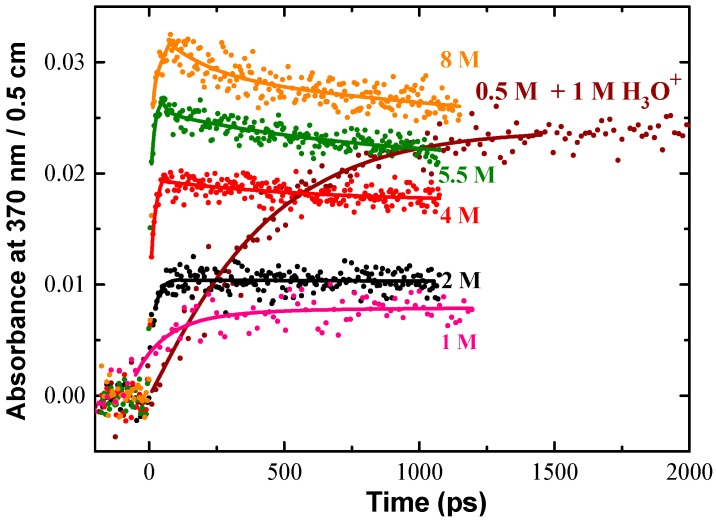
Transient kinetics observed at 370 nm in a wide range of NaCl aqueous solutions to show the products of oxidation of Cl^−^. The absorbance of hydrated electron and transient signal from irradiated fused silica were subtracted from the raw data. The solid lines are drawn to guide the eye. The absorbed dose (22.5 Gy per pulse) was the same for all kinetics. The acid used to adjust the pH of solution was HClO_4_. Adapted from [37].

As an example, in Cl^−^ aqueous solution, the transient products of radiation-induced oxidation of Cl^−^ ions are observed at 370 nm, as shown in Figure 4. The following reactions are considered:

(2) $$Cl^{-}\to Cl^{•}+e^{-}$$

(3) $$OH^{•}+Cl^{-}\to ClOH^{•-}$$

(4) $$ClOH^{•-}\to Cl^{-}+OH^{•}$$

(5) $$Cl^{•}+Cl^{-}\to Cl_{2}^{•-}$$

(6) $$Cl^{-}+H_{2}O^{•}{}^{+}\to Cl^{•}+H_{2}O$$

In acidic solutions, we have the following fast proton reaction:

(7) $$ClOH^{•}{}^{-}+H^{+}\to Cl^{•}+H_{2}O$$

Reaction (2) is due to the direct effect that means one-electron oxidation of Cl^−^ by ionizing radiation. It occurs when the number of electron of the solute in the solution is not negligible compared to that of water. It happens when the concentration is higher than 0.5 M. Reaction (3) is issued from the water radiolysis. That reaction is not complete because Reaction (4) takes place. Reaction (5) is controlled by diffusion, and finally the electron transfer in Reaction (6) is ultrafast and can occur if the radical cation of water is formed in contact of Cl^−^. The initial absorbance reported in Figure 4 corresponds to the formation of ClOH^•−^ and Cl_2_^•−^ radicals both absorbing around 370 nm, but with different extinction coefficient. ClOH^•−^ radicals are produced by an equilibrium with OH^•^ scavenging reaction and Cl_2_^•−^ is generated from direct ionizing of Cl^−^ and probably from H_2_O^•+^ oxidation followed by a fast reaction of Cl^•^ with Cl^−^. As the extinction coefficient of each species at 370 nm is well-known, the yield of the radicals can be analyzed from the kinetics. The kinetics simulations show the amount of Cl_2_^•−^ formation within the electron pulse increases notably at an increasing Cl^−^ concentration. It also reveals that the direct ionization of Cl^−^ cannot solely explain the significant amount of fast Cl_2_^•−^ formation at short timescale. When the acid is used, ClOH^•−^ is converted into Cl^•^ (7); one can readily see in Figure 4 that the total absorption in 0.5 M acid solutions very nearly matches that for 8 M Cl^−^ neutral solutions where the OH^•^ radical scavenging capacity is much higher [37].

This difference between acid and neutral solutions is shown in Figure 4 for 0.5 M Cl^−^ solutions. Conversion of the ClOH^•−^ is relatively slow in acid solutions, but it is total and occurs at greater extent than in neutral solutions.

**Figure 5:**
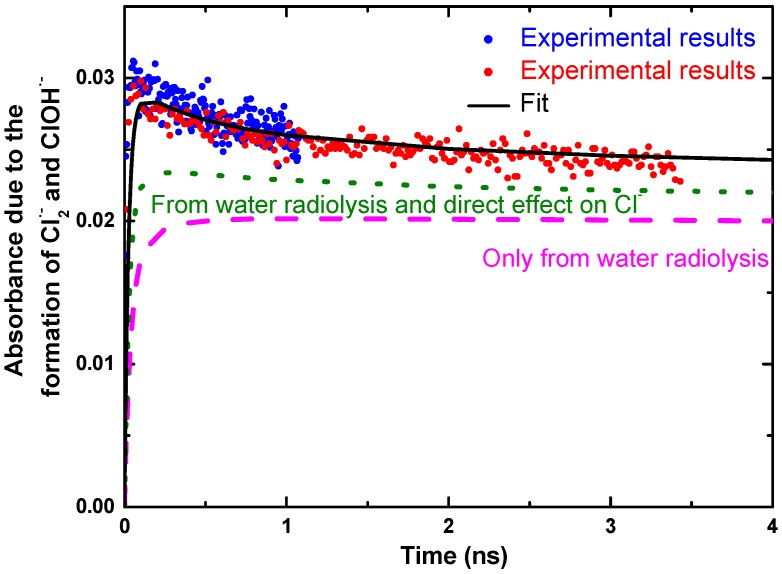
Observed absorbance decay (blue and red data for short and long time scale) in solution containing 5.5 M NaCl with model simulations of spur reactions: (dashed line) simple water model alone without any direct effect; (dotted line) with direct ionization of Cl^−^; (solid line) with direct ionization of Cl^−^ and 30% scavenging of H_2_O^•+^ by Cl^−^. The dose was 22.5 Gy per pulse. Adapted from [37].

The predictions of the diffusion kinetic modeling of the spur for solutions containing 5.5 M Cl^−^ are shown in Figure 5. The predicted yields for just the Cl_2_^•−^ radical alone are also shown in Figure 5. The standard water radiolysis model gives a much slow products formation and predicts a maximum that is considerably lower than the observed absorbance. The direct ionization of Cl^−^ to give Cl^•^ and e_aq_^−^ in 5.5 M Cl^−^ solutions can be included by assuming 0.24 percent of the energy is deposited directly into the Cl^−^. Again, the model predictions are too slow and the maximum is lower than the observed absorbance. Clearly, the only method to the increase production of ClOH^•−^ and Cl_2_^•−^ radicals on the short time scale is to have a scavenging process for Cl^−^ reaction with a water transient species. Inclusion of the reaction of Cl^−^ with H_2_O^•+^ increases the predicted absorbance with faster rise times. For 5.5 M Cl^−^ solution, almost 30% of the H_2_O^•+^ must be scavenged by Cl^−^ to match the observed absorbance measurements.

Therefore, it is concluded that the precursor of the OH^•^ radical, i.e., H_2_O^•+^ radical, forms Cl^•^ atom within the electron pulse and the Cl^•^ atom reacts subsequently with Cl^−^ to form Cl_2_^•−^ on very short time scales. It is important to note that the reduction potential of Cl^−^ is very high and only a powerful oxidizing species can transform Cl^−^ into Cl^•^.

## 4. Reactivity of H_2_O^•+^ in Highly Acidic Solutions

**Figure 6:**
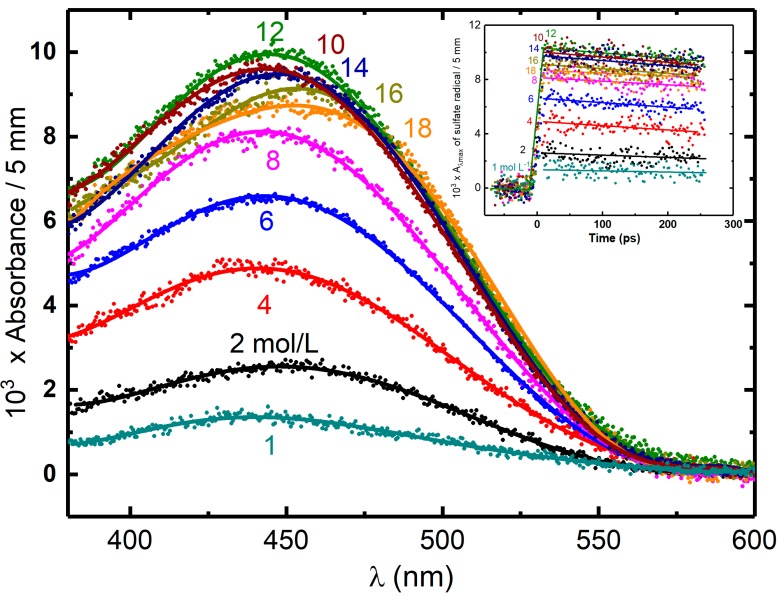
Transient absorption spectrum of SO_4_^•−^ or HSO_4_^•−^ at maximum of absorption versus concentration of acid observed immediately after 7 ps electron pulse. Insert figure shows the corresponding transient kinetics of SO_4_^•−^ or HSO_4_^•−^. The contribution of solvated electron in 1 to 6 mol L^−^^1^ H_2_SO_4_ is subtracted from the signal based on the known extinction coefficient at various wavelengths. The energy deposited in concentrated acidic solutions *D_sol_* is higher comparing with neat water because of direct radiolysis of the solutes in the presence of the system. The effective dose considering this contribution can be derived from the reference dose in pure water *D_water_* as follows: $D_{sol}(JL^{-1})=FD_{water}(Jkg^{-1})$ The dose factor (*F*) can be estimated by the following equation: $F=d_{sol}(Z_{SO_{4}^{2-}}p/A_{H_{2}SO_{4}}+Z_{water}(100-p)/A_{water}){\left(Z_{water}100/A_{water}\right)}^{-1}$, where $d_{sol}$ is the density of the solution, *Z* is the number of electrons, *A* is the mass number, and *p* is the weight fraction of the solute percent. The dose was 31.3 Gy per pulse. Adapted from [43].

Unfortunately, as the yield of the secondary radicals from direct ionizing is not quantitatively known, the exact radiolytic yield and reactivity of H_2_O^•+^ in various concentrations remain to be elucidated. Sulfuric acid solution constitutes an ideal system to verify the hypothesis and to investigate the reactivity of H_2_O^•+^ due to several reasons. First, it is a homogenous phase without molecules clustering, which guarantees the sufficient close encounters (H_2_O^•+^ … SO_4_^2–^) [40]. Second, the yield of SO_4_^•−^ from the direct effect can be measured from the almost neat sulfuric acid (18 M) and it is possible to correlate this contribution to various concentrations by using the factor of the electron fraction. Third, the rate constant of the reaction of OH^•^ radical with SO_4_^2−^ is relative low (10^8^ L^−1^ mol cm^−1^) and the reaction time is several nanoseconds at even the highest concentration [41]. Fourth, the counter ion of acid is H^+^ without valence electron. Thus, unlike the salt ions such as Na^+^ or Mg^2+^, the direct ionizing of H^+^ itself does not yield to any oxidizing species. The last reason is that the spectrum and the molar extinction efficiency (1600 cm^−1^ M^−1^) of secondary radicals SO_4_^•–^ are well-known, so the yield is ready to be deduced [42]. Figure 2 presents the schematic description of the possible reactions occurring in the sulfuric acid solutions at *sub*picosecond time scales involving proton transfer (t. p^+^, black), direct ionizing of solutes SO_4_^2−^ (red), ultrafast electron transfer (t. e^−^, blue) and electron relaxation. To clarify all of these processes, we performed the picosecond pulse radiolysis of the solutions containing a wide range of sulfuric acid concentration [43]. Figure 6 displays the absorption spectrum of SO_4_^•–^ in a variety of sulfuric acid solutions ranging from 1 M up to 18 M observed on the ps timescale (immediately after 7 ps electron pulse). It has a typical absorption band at 450 nm. The shape of this band does not depend to the concentration and no significant change in extinction coefficient was found in the concentrated sulfuric acid as previous reported [40]. It only slightly shifts to higher wavelength in response to higher concentration owning to the acid equilibrium of SO_4_^•–^ to HSO_4_^•^. As it is known the SO_4_^•–^ formation is not corresponding to OH^•^ radical at this observed timescale, only two possible pathways accounts for producing SO_4_^•–^radicals, that is direct ionizing and ultrafast electron transfer from H_2_O^•+^. In Figure 4, it can be seen that, after dose correction, the absorbance of SO_4_^–•^ in relation with its radiolytic yield is increasing with the sulfuric concentration increasing from 1 M to 12 M. This can be understood because the direct ionizing becomes more important as more solutes in present. Interestingly, from 12 M to 18 M, as water molecule is not abundant, the absorbance continuously drops. These observations clearly present an evidence that direct ionizing alone cannot be the only effect, interpreting the formation of SO_4_^•–^ is due to at least two pathways, otherwise the absorption would keep increasing. There must exist an ultrafast process of H_2_O^•+^ that occurs on the timescale of femtosecond, accounting for the supplementary yield of SO_4_^•–^. The radiolytic yield of SO_4_^•–^ is directly deduced to be 3.75 × 10^–7^ molJ^–1^ from the 18 M sulfuric acid. Based on the electron fraction of solutes in solutions, the yield contribution of direct and indirect effect can be obtained correspondingly as following equation.

(8) $$G_{\exp}=f_{s}G_{\mathrm{dir}}+f_{w}G_{in\mathrm{dir}}$$

where *G_dir_* is the yield of direct ionization of solutes, and *f_s_* and *f_w_* represent the fraction of solute and water electron density, respectively. This parameter indicates the ratio of the energy directly absorbed by the solute *S*. The yield of reaction produced by the water radical cation is denoted *G_indir_*.

**Figure 7:**
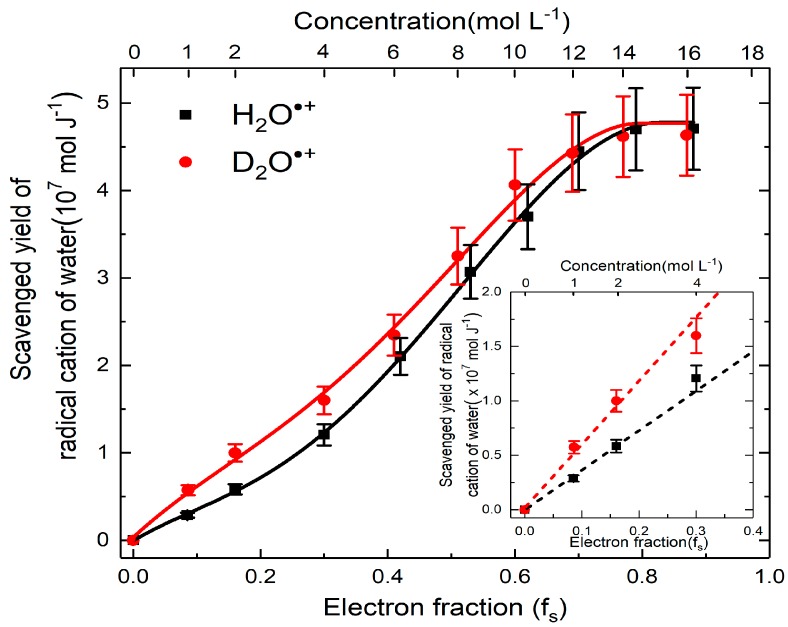
Yields of H_2_O^•+^ scavenging versus concentration case of H_2_SO_4_ and D_2_SO_4_. The difference between H_2_SO_4_ and D_2_SO_4_ are zoomed in insert figure to better present the isotope effect in proton transfer.

From the above analysis, the yield of H_2_O^•+^, which participates in ultrafast oxidation is deduced and their values are plotted as a function of concentration correlated with the electron fraction. The analyzed results in Figure 7 show four striking characteristics. First, at very low concentration or in dilute medium, neither direct effect nor H_2_O^•+^ oxidation is important as the formation of SO_4_^•–^ is not observed. Second, the yield of H_2_O^•+^ is highly dependent on the concentration, particularly below 12 M, where we observed a continuous increasing trend with the rising of the solute concentration. This is in agreement with our predications and it suggested the probability of electron transfer of H_2_O^•+^ is greater at higher concentration. Third, after 12 M, the yield is saturated to (4.8 ± 0. 5) × 10^−7^ mol J^−1^, which is found to be similar with the value of OH^•^ radical yield reported at picosecond timescale in neat water [44]. In these cases, it can be concluded that after certain concentration, all of the precursors of OH^•^ react with sulfuric solutes. Fourth, a remarkable difference between the yield of the sulfate radical in deuterated and hydrogenated solutions is observed at lower concentration (Figure 7). Density functional theory simulations suggests the electron transfer of H_2_O^•+^ proceeds via sub-femtosecond charge migration and is not affected by isotopic substitution [45]. The proton transfer is affected by isotopic effect: it is slowed down because the vibration mode which is involved in proton transfer reaction depends to the mass. Therefore, by observing more efficient electron transfer reaction in heavy water than in H_2_O (Figure 7), it is demonstrated that the oxidation triggered is in competition with the proton transfer reaction.

In analogy to H_2_SO_4_ system, H_3_PO_4_ solutions also have the above-mentioned advantages, so the picosecond pulse radiolysis measurements were further performed in the phosphate system [46]. The acidic form of phosphate radicals (H_2_PO_4_^•^) exhibiting an absorption peak at 520 nm with a molar extinction coefficient of 1850 L mol^−1^ cm^−1^ is the indicator of direct and H_2_O^•+^ oxidation. The overall trend is found to be similar, whereas the saturated yield of H_2_O^•+^ is significantly lower comparing with that in sulfuric acid solution. The reasons to explain why the water hole trapping in phosphoric acid is not efficient are not clear. However, based on the microscopic structure of phosphate solutions from X-rays diffraction measurements [47,48], we may propose that the existence of aggregations of phosphates should be taken into account for this. Because of the formation of dimer or trimer at higher concentrations, the amount of close encounters between water and phosphate is relatively less than those in sulfuric acid under identical molar concentration, and thus the efficiency of in situ H_2_O^•+^ capturing reaction is lower.

Although there have been studies of H_2_O^•+^ reactivity towards HNO_3_, H_2_SO_4_ and H_3_PO_4_, there are two additional acids (perchlorate acid and hydrofluoric acid) remaining that are interesting in aqueous system. Unfortunately, direct ionizing of perchlorate acid gives rise to two components of radicals (ClO_4_^•^ and ClO_3_^•^) with very low value of extinction coefficient, so it might not be easy to correctly deduce the secondary radical yield from H_2_O^•+^. The fact that the element fluorine has a high electron affinity may result in a high value of redox potential of one electron couple F^•^/F^–^, which has not been known yet. It is of great interest to testify the oxidizing ability of H_2_O^•+^ by observing the formation of F^•^ or F_2_^•−^ in pulse radiolysis of highly concentrated HF solution. This measurement has not been performed so far because it requires a very specific fused cell and solution cycling system to avoid the corrosions from HF samples and safety issues when handling with HF.

One may assume that H_2_O^•+^ is, in fact, the acidic form of OH^•^ in aqueous solutions but the p*K*_a_ value is not clear. It is known OH^•^ radical will attack guanine molecules, the most easily oxidized DNA bases, through a series of complex processes. It involves H-abstraction, adducting and one-electron oxidation reaction at similar rates, leading to the formation of a variety of guanine based radicals. Interestingly, our recent study of radiation-induced guanine oxidation in highly concentrated phosphoric acid (6 M) shows all of OH^•^ radicals in strong acid condition solely oxidize guanine molecule to guanine cation (G^•+^). It is therefore inferred that H_2_O^•+^ radical cation in some cases indeed displays a distinguished reactivity from OH^•^ radical. Besides, we are carrying out pulse radiolysis measurements of highly concentrated DNA subunits solutions in order to extend our findings obtained in inorganic solutions to a situation that is much biologically relevant.

## 5. The Nature and Ultrafast Charge Migration of H_2_O^•+^

Although the 7 ps electron pump-probe measurements have revealed the chemical reactivity of H_2_O^•+^, the investigations on charge migration of H_2_O^•+^ in liquid water (proton transfer to OH^•^ radical) are still not sufficient to give a definitive conclusion. In 1990, Eisenthal et al. [49] observed an ultrafast rise and decay (˂60 fs) when the probe light of 625 nm was used in a femtosecond pump-probe study of hydrated electron. They believed this visible absorption signal is attributed to H_2_O^•+^ rather than the excited states of H_2_O because H_2_O* absorbs at longer wavelength. Gauduel et al. [50] also reported using the ultraviolet femtoseconds pulses photoionization, that this water radical cation presents a transient absorbance in the ultraviolet spectral domain (460~410 nm). The evolution time of this ion molecule reaction was estimated as 100 fs in light water against 170 fs in heavy water. However, the charge localization step of H_2_O^•+^ towards water molecules is suggested to be less than 40 fs by a theoretical simulation. Besides, to reexamine this issue, a subsequent transient absorption experiment using a 30 fs pulse with the energy of 11 eV was carried out by Marsalek et al. in the same work. However, they did not see any transient optical signature of reaction in the visible and deep UV. It was then argued that the femtosecond photolysis used by Gauduel et al. at the pump energy of 8 eV is, in fact, not sufficient to generate the H_2_O^•+^ directly. The induced absorbance at 100 fs is most likely to be the simultaneous absorption of one pump and one probe when the two laser pulses are overlapped in time.

Nevertheless, very recently, a polarization anisotropy measurement by Li et al. estimated the lifetime of this transient species to be as long as 195 ± 5 fs [51]. In all of the above experiments, H_2_O^•+^ is defined as a water molecule with an electron removed from its valence shell. In addition to this, X-ray core-level ionization of liquid water suggested a proton-transfer dynamics occur on the same timescale as electron autoionization [52]. In this study, proton transfer forms a Zundel-type intermediate [HO*···H···H_2_O]^•+^, which further ionizes to form a so-far unnoticed type of dicationic charge-separated species with high internal energy. On the other hand, the recent density functional theory simulations decipher the electron transfer mechanism of H_2_O^•+^: it proceeds via sub-femtosecond charge migration in competition with the proton transfer and is not affected by isotopic substitution [45].

From the above conflicting results, it is of great interest to explore and understand the nature of H_2_O^•+^ by using a more sophisticated time-resolved experimental or theoretical method. We note several points that should be taken into account. First, from the photoelectron spectroscopy of liquid water, it is known that the minimum energy required for generate a photoelectron is 11.16 eV. Owning to a broad conduction band of liquid water, hydrated electron can also be produced by photolysis with the pump energy well below 11 eV or by the two-photoionization. Thus, depending on the energy of radiation deposited, the hole radical may exists as an electron-radical complex [H_3_O^+^e_hyd_^−^…OH^•^] or a H_2_O^•+^ with a distance far from the excess electron [53,54]. Second, the simulations indicate the molar extinction coefficient for localized H_2_O^•+^ is crudely predicated as 80–100 M^−1^ cm^−1^ at peak and a blue shift from 540 nm to 200 nm at the initial stage (<100 fs) is expected to occur as H_2_O^•+^ relax to OH^•^ radical. In this case, probing H_2_O^•+^ by transient absorption spectroscopy in UV region will be challenging. The observation of dynamic is suggested to be made in the formation of OH^•^ radical in heavy water. In addition to this method, attosecond photoelectron spectroscopy based on the high harmonic generation with higher energy pulse (20 eV–100 eV) is recently applied on liquid water phase, which shows potential ability to resolve H_2_O^•+^ [55].

## 6. Perspectives on Reactivity of H_2_O^•+^ in Other Environments

**Figure 8:**
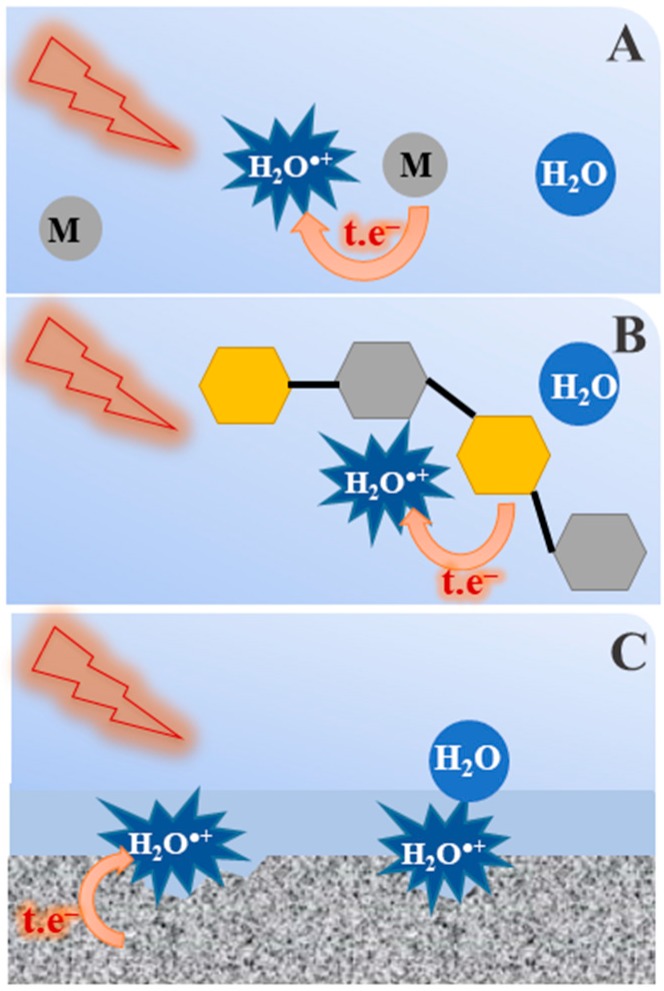
Under certain conditions, irradiated water can form a water radical cation that undergoes an electron transfer reaction in competition with the proton transfer reaction: (**A**) concentrated solutions in which the first solvation shell is occupied by solute molecules or ions; (**B**) water layers in contact with biomolecules; and (**C**) water/solid interface.

In addition to irradiation of concentrated H_2_SO_4_ or H_3_PO_4_ solutions, the direct oxidation by the H_2_O^•+^ is expected to take place in other chemical environment where the probabilities of its nearest neighbors being H_2_O or another molecule. As shown in Figure 8, some examples are given below:

- Radiotherapy and radiobiology: It is known that large amounts of hydrating water molecules are in direct contact with biomolecules such as DNA or proteins [56]. When ionizing radiation is present, part of the radiation energy is absorbed directly by DNA and breaks the bonds of the sugars, phosphates and nucleobases, while some of the radiation is also absorbed by the water adjacent to the DNA. In fact, some amount of water is linked to the DNA and the local concentration of DNA is very high. In that case, the generated H_2_O^•+^ radical cation may induce a chemistry different from OH^•^ radical and the consequence is producing the secondary biomolecule radicals which has not been yet considered before.
- Treatment and storage of fuel in the nuclear industry: Spent nuclear fuel is processed in highly concentrated nitric acid aqueous solutions. In this case, the radical cation H_2_O^•+^ may react with nitrate ions to yield the NO_3_^•^ radical, which is also a highly oxidizing species. Alternatively, when radioactive waste of low and medium level is coated by cement or any other porous material, the interface effect is extensive and the formation of radical cation H_2_O^•+^ and its oxidation reactions should be taken into account.
- When the core of a nuclear power plant comes into contact with water, as happened during the Fukushima incident in Japan, the amount of radiation deposited at the interface of the exposed fuel/water is important. Although we do not provide any evidence, the present work suggests that, in this situation, metal corrosion by H_2_O^•+^ may be involved [57].

For the above reasons, the investigation on the properties of this ultrashort lived radical cation is very important. However, we need to respond to the following questions and challenges:

- Is the positive charge distributed on several water molecules or is it localized mainly in one water molecule? H_2_O^•+^ symbol stands for the water radical cation, but is it possible that the positive charge is distributed on several water molecules (H_2_O^•^)^+^_n_?
- The absorption band of the water radical cation is not known, but its deprotonated form, OH^•^ radical, absorbs in the region of 200–300 nm. Thus, it is rationalized that ultrafast detection setup (˂30 fs) is needed to observe in deep UV region to confirm that the decay of this species is correlated to the formation of OH^•^ radical in neat water.
- What is the time constant for the decay of H_2_O^•+^? This value is important to consider for the competition between the electron transfer and the proton transfer reactions of H_2_O^•+^.
- Finally, more precise estimation is needed to evaluate the oxidizing power of H_2_O^•+^. The redox potential and the *pKa* of this radical cation should get better estimation.
